# Perinatal outcomes associated with ICP in twin pregnancies were worse than singletons: an almost 5-year retrospective cohort study

**DOI:** 10.1186/s12884-022-05160-6

**Published:** 2022-11-05

**Authors:** Tingting Xu, Chunyan Deng, Yongchi Zhan, Qing Hu, Guiqiong Huang, Xing Wang, Bing Guo, Haiyan Yu, Xiaodong Wang

**Affiliations:** 1 Department of Obstetrics and Gynecology, West China Second University Hospital, Sichuan University, Chengdu, Sichuan China; 2 Key Laboratory of Birth Defects and Related Diseases of Women and Children (Sichuan University) Ministry of Education, Chengdu, 610041 Sichuan China; 3 West China School of Public Health and West China Fourth Hospital, Sichuan University, Chengdu, Sichuan China

**Keywords:** Intrahepatic cholestasis of pregnancy, Twin pregnancies, Retrospective cohort study, Perinatal outcomes

## Abstract

**Background:**

Intrahepatic cholestasis of pregnancy (ICP) is associated with an increased risk of adverse perinatal outcomes leading to high perinatal morbidity and mortality. However, few studies have examined twin pregnancies complicated by ICP. To assess the perinatal outcomes of twin pregnancies with ICP, a retrospective cohort study was conducted.

**Methods:**

A total of 633 twin pregnancies and 1267 singleton pregnancies with ICP were included. In addition, a correlation study was performed on the matched total bile acid (TBA) levels from maternal serum, fetal umbilical venous blood, and amniotic fluid of 33 twin pregnancies from twin groups.

**Results:**

When compared to singletons, twin pregnancies with ICP had a higher risk of cesarean section (CS) (96.4% vs. 76.1%), preterm birth (PTB) (82.6% vs. 19.7%), fetal distress (2.0% vs. 1.3%), and neonatal intensive care unit (NICU) admission (23.6% vs. 5.1%), which was significantly related to increasing TBA levels (*P <* 0.05). In twin pregnancies with TBA ≥100 μmol/L, the incidences of CS, PTB, fetal distress, neonatal asphyxia, and meconium-stained amniotic fluid were 94.4, 100, 11.1, 5.6, and 36.1%, respectively. Furthermore, the maximum maternal TBA levels were positively correlated with TBA levels in the amniotic fluid (*r =* 0.61, *P <* 0.05) and umbilical cord blood (*r =* 0.44, *P <* 0.05), and a similar correlation was found for maternal TBA levels at delivery. TBA levels in umbilical cord blood and amniotic fluid also had a significant and positive correlation (*r =* 0.52, *P <* 0.05).

**Conclusions:**

Twin pregnancies with ICP had a higher risk for adverse perinatal outcomes than singletons, which was associated with higher TBA levels. TBA can be transported through the placenta and is involved in uterus-placenta-fetal circulation.

## Background

Intrahepatic cholestasis of pregnancy (ICP) is characterized by maternal persistent pruritus, increased serum total bile acid (TBA) concentrations, and elevated serum liver aminotransferase activity [1]. It usually presents in the late second or third trimesters [1–3]. ICP has a wide range of prevalence, ranging from 0.2 to 25% [4–6]. However, the prevalence of ICP in multiple pregnancies is five times greater than that in singleton pregnancies [3], and the prevalence of ICP in twin pregnancies is approximately 20-25% [7, 8].

ICP is associated with an increased risk of several adverse perinatal outcomes, including spontaneous and iatrogenic preterm birth (PTB), meconium-stained amniotic fluid (MSAF), fetal distress, fetal hypoxia, sudden intrauterine fetal death, respiratory distress syndrome (RDS), neonatal asphyxia, and neonatal intensive care unit (NICU) admission [1, 3, 7, 9–16], which lead to a higher risk of perinatal morbidity and mortality. Hence, active management and close antenatal monitoring are urgently needed for these pregnant women [17].

Due to an earlier presentation and higher maternal serum TBA levels, ICP appears to be more severe in twin pregnancies than in singletons [7]. However, universal or standard recommendations or professional consensus on the prenatal management of twin pregnancies with ICP are lacking. Hence, we conducted a nearly five-year retrospective cohort analysis of twin pregnancies with ICP in our hospital. We hope our findings will provide some evidence to guide the available options for their clinical treatment.

## Methods

### Study design overview

All included patients received routine prenatal care and were born at our hospital from Jan. 1, 2016 to Jun. 30, 2020. All data were abstracted from the electronic medical records system.

### Inclusion criteria and exclusion criteria

ICP is characterized by maternal serum TBA ≥ 10 μmol/L, with or without maternal pruritus, that completely normalizes after delivery in the absence of other causes of liver dysfunction or itching [18]. In addition, ICP is diagnosed in cases of maternal pruritus with elevated serum liver transaminase enzymes and normal TBA levels (TBA < 10 μmol/L); specifically, alanine aminotransferase (ALT) and aspartate aminotransferase (AST) levels are twice the normal upper limits [11, 18, 19].

The gestational age of all included cases of ICP was estimated based on the first-trimester ultrasound scan. Furthermore, the chorionicity and amnionicity of twin pregnancies with ICP were detected by ultrasonography in the first trimester and confirmed by postnatal pathology.

### Main outcome*s*

Maternal age, height, weight gain throughout the pregnancy, family history of ICP, history of ICP, in vitro fertilization and embryo transfer (IVF-ET), PTB (iatrogenic and spontaneous), gestational age at delivery, cesarean section (CS), vaginal delivery (VD), MSAF, fetal distress, stillbirth, neonatal birth weight and height, neonatal asphyxia, neonatal death, and NICU admission were included. According to Rodrguez Fernández V, et al. [20], and different degrees of MSAF (***yellow***, ***green,*** and ***thick***) were employed to compare to clear amniotic fluids in our investigation.

### Laboratory analysis

The matched maternal serum, umbilical venous blood, and amniotic fluid samples of 33 twin pregnancies from twin groups were collected during the cesarean section for TBA tests. The maternal serum and umbilical cord blood levels of alanine transaminase (ALT, U/L), aspartate transaminase (AST, U/L), TBA (umol/L), total bilirubin (TBIL, umol/L), direct bilirubin (DBIL, umol/L), indirect bilirubin (IDIL, umol/L), γ-glutamyltransferase (GGT, U/L), albumin (ALB, g/L), globulin (GLB, g/L), albumin/globulin (A/G), total cholesterol (TC, mmol/L), triglyceride (TG, mmol/L), high-density lipoprotein (HDL, mmol/L), low-density lipoprotein (LDL, mmol/L), alkaline phosphatase (ALP), lactate dehydrogenase (LDH, U/L), prealbumin (PA, mg/L), fasting plasma glucose (FPG, mmol/L), apolipoproteins A1 (apo A1, g/L), apolipoproteins B (apo B, g/L), homocysteine (HCY, umol/L), sodium (Na), phosphorus (P, mmol/L), magnesium (Mg, mmol/L), kalium (K, mmol/L), chlorine (Cl, mmol/L), and calcium (Ca, mmol/L) were detected by Siemens-advia® chemistry kits using a fully automatic biochemical analyzer (Siemens-advia® 2400, Germany) at a medical laboratory of West China Second University Hospital of Sichuan University.

### Perinatal management of ICP

Regular antenatal care for ICP was performed every 2 weeks before 28 weeks and every 1 week after 28 weeks. These prenatal tests included a nonstress test (NST), antenatal ultrasound scanning, and serial biochemical surveillance to detect worsening conditions and the risks to the fetus. Usually, NST begins at the 32nd gestational week, and it was recommended once per week for mild ICP and twice per week for severe ICP. The amniotic fluid volume, umbilical artery Doppler (S/D ratio), and fetal growth and development were all assessed using serial ultrasonography. Maternal serum liver function and TBA levels were dynamically monitored using a serial biochemical examinations. Prenatal corticosteroids were indicated for pregnant women with ICP who were at risk of PTB to enhance fetal lung development, as well as intramuscular injections of vitamin K to prevent intraventricular hemorrhage (IVH) in neonates with ICP. If fetal hypoxia or adverse events were recognized, an emergency cesarean section was immediately performed.

### Statistical analysis

The data were compiled and examined using the R programming language (version 3.5.0; R Foundation for Statistical Computing, Vienna, Austria). For a baseline comparison, continuous variables were analyzed with medians and interquartile ranges (IQRs), whereas categorical variables were analyzed based on numbers and percentages. The rank-sum test was used to compare the distribution of quantitative data that did not conform to the normal distribution with the T test. For continuous variables, the mean ± standard deviation (SD) was computed for each group, and relative frequencies were estimated for categorical variables using the chi-square test (χ2 test). Multiple logistic regression models were used to obtain crude and adjusted odds ratios (ORs) for ICP with 95% confidence intervals (CIs). The correlation matrix was built using the Pearson parametric correlation test. The Holm correction was used to alter the probability values of correlation matrices for various measures. To evaluate the correlation of TBA levels, Pearson correlation scatterplots were constructed. All statistical tests were performed using a two-sided test, with significant differences defined as *P <* 0.05.

## Results

A total of 633 twin pregnancies and 1267 singleton pregnancies with ICP were studied. The composition of twins includes 2 monochorionic monoamniotic (MCMA), 136 monochorionic diamniotic (MCDA), and 495 dichorionic diamniotic (DCDA) twin pregnancies. A significant difference was found in weight gain throughout pregnancy (12 vs. 15 kg), the incidence of primiparity (67.6% vs. 84.2%), ICP in a previous pregnancy (1.3% vs. 6.5%), IVF-ET (6.7% vs. 58.6%), and clinical symptoms (56.6% vs. 59.2%) between singleton and twin pregnancies with ICP (*P <* 0.05). Similarly, gestational weeks at delivery (37.44 ± 1.68 vs. 35.07 ± 1.71) also significantly differed between these two groups (Table 1).

**Table 1:** Clinical characteristics of the included population

Group	Singleton	Twin	P
N	1267	633	
Age [years], mean (IQR)	30 (28, 34)	31 (28, 34)	0.51
Height [cm], mean (IQR)	160 (156, 162)	160 (156, 163)	0.66
Weight gain in pregnancy [kg], mean (IQR)	12 (10, 15)	15 (11, 18)	< 0.05
Gravidity, mean (IQR)	2 (1, 3)	2 (1, 3)	< 0.05
Parity, mean (IQR)	1 (1, 2)	1 (0, 1)	< 0.05
Primiparous, n (%)	856 (67.6)	533 (84.2)	< 0.05
ICP in previous pregnancy, n (%)	16 (1.3)	41 (6.5)	< 0.05
Family history of ICP, n (%)	0 (0.0)	0 (0.0)	NaN
IVF-ET, n (%)	85 (6.7)	371 (58.6)	< 0.05
Clinical symptoms, n (%)	716 (56.6)	375 (59.2)	< 0.05
Gestational weeks at time of diagnosis [week], mean ± SD	16.80 ± 8.08	17.40 ± 7.72	0.13
Gestational weeks at delivery [week], mean ± SD	37.44 ± 1.68	35.07 ± 1.71	< 0.05

*IQR* Inter quartile range, *IVF-ET* In vitro fertilization-embryo transfer, *ICP* Intrahepatic cholestasis of pregnancy, Clinical symptoms including pruritus, itch, jaundice, anorexia, etc

The incidences of CS (96.4% vs. 76.1%), PTB (82.6% vs. 19.7%), fetal distress (2.0% vs. 1.3%) and NICU admission (23.6% vs. 5.1%) significantly differed between twin pregnancies and singleton pregnancies with ICP (*P <* 0.05). The mean neonatal birth weight was significantly lower in twins with ICP than in singleton pregnancies with ICP (2248.28 ± 416.20 vs. 3043.69 ± 509.10 g, respectively, *P <* 0.05), while the mean neonatal birth height was significantly lower in twins than in singleton pregnancies with ICP. The mean gestational age was significantly earlier for twins than for singleton pregnancies (35.07 ± 1.71 vs. 37.44 ± 1.68 weeks, respectively, *P <* 0.05). Subjects who had PTB were divided into three categories (< 37 weeks, < 34 weeks, < 32 weeks) according to gestational age. Most of the fetuses of twin pregnancies with ICP (82.6%, 1046/1266) were born before 37 weeks. (Table 2).

**Table 2:** The perinatal outcomes of twin and singleton pregnancies with ICP

	singleton	twins	OR(95%CI)	P	Adjusted OR(95%CI)	Adjusted P
**Obstetric outcomes**	1267	633				
CS, n (%)	964 (76.1)	610 (96.4)	8.34 (8.02, 8.66)	< 0.05	7.36 (7.03, 7.69)	< 0.05^*^
VD, n (%)	303 (23.9)	23 (3.6)	0.12(− 0.201, 0.441)	< 0.05	0.14(− 0.19, 0.47)	< 0.05^*^
MSAF, n (%)	159 (12.5)	85 (13.5)	1.09 (0.86, 1.32)	0.47	1.23 (0.99, 1.48)	0.09
**Neonatal outcomes**	1267	1266				
Gestational age [weeks] (mean ± SD)	37.44 ± 1.68	35.07 ± 1.71	–	< 0.05	–	< 0.05^*^
Preterm birth < 37 weeks (%)	250 (19.7)	1046 (82.6)	0.23(− 0.38, 0.84)	< 0.05	0.15(− 0.49, 0.79)	< 0.05^*^
Preterm birth < 34 weeks (%)	38 (3.0)	186 (14.7)	0.18(−0.18, 0.54)	< 0.05	0.15(− 0.23, 0.53)	< 0.05^*^
Preterm birth < 32 weeks (%)	13 (1.0)	55 (4.3)	0.05(− 0.15, 0.25)	< 0.05	0.04(− 0.17, 0.26)	< 0.05^*^
Birth height (cm), (mean ± SD)	48.53 ± 2.44	45.18 ± 2.70	–	< 0.05	–	< 0.05^*^
Birth weight (g), (mean ± SD)	3043.69 ± 509.10	2248.28 ± 416.20	–	< 0.05	–	< 0.05^*^
Neonatal asphyxia, n (%)	13 (1.0)	19 (1.5)	1.48 (0.77, 2.19)	0.28	0.53(−0.32,1.38)	0.15
Fetal distress, n (%)	17 (1.3)	25 (2.0)	1.49 (0.87, 2.11)	0.21	0.30(−0.44, 1.05)	< 0.05^*^
Stillbirth, n (%)	0 (0.0)	5 (0.4)		0.99		0.99
Neonatal death, n (%)	1 (0.1)	0 (0.0)		0.99		0.98
NICU, n (%)	65 (5.1)	297 (23.6)	5.70 (5.42, 6.0)	< 0.05	1.9 (1.53, 2.27)	< 0.05^*^

*ICP* Intrahepatic cholestasis of pregnancy, *TBA* Total bile acid, *CS* Cesarean section, *VD* Vaginal delivery, *PTB* Preterm birth, *NICU* Neonatal Intensive Care Unit, *MSAF* Meconium-stained amniotic fluid

In total, 618 twin pregnancies among the 633 twin pregnancies with ICP has a TBA ≥10 μmol/L. The maximum TBA level could determine the severity of ICP [8, 21]. To undertake subgroup analyses, these pregnant women were stratified into three categories based on the maximum level of TBA throughout the pregnancy: 10 ≤ TBA < 40 μmol/L, 40 ≤ TBA < 100 μmol/L, and TBA ≥100 μmol/L [22, 23]. Significant differences were found in the risk of CS, PTB, stillbirth, fetal distress, neonatal asphyxia, birth height, birth weight, and gestational age between these three groups (*P <* 0.05). The incidences of CS, PTB, stillbirth, fetal distress, neonatal asphyxia, and MSAF increased as TBA concentration increased. In twin pregnancies with TBA ≥100 μmol/L, the incidences of CS, PTB, fetal distress, neonatal asphyxia, and MSAF were 94.4, 100, 11.1, 5.6, and 36.1%, respectively. All fetuses of twin pregnancies with TBA ≥100 μmol/L were born before 36 weeks, with median gestational ages of 36 (IQR 34 to 36 weeks), 35 (IQR 34 to 36 weeks), and 34 (IQR 33 to 35 weeks) among these three groups (*P <* 0.05). The percentage of low birth weight in twin pregnancies with TBA ≥100 μmol/L was 94.4. (Table 3).

**Table 3:** The subgroup analysis of perinatal outcomes of twin pregnancy of ICP with TBA ≥10 umol/L

Indicator	TBA [10-40)	TBA [40-100)	TBA ≥ 100	p
***obstetric outcomes***	459	141	18	
CS, n (%)	449 (97.8)	129 (91.5)	17 (94.4)	< 0.05*
VD, n (%)	10 (2.2)	12 (8.5)	1 (5.6)	< 0.05*
PTB, n (%)	364 (79.3)	129 (91.4)	36 (100.0)	< 0.05*
Gestational age [week], median (IQR)	36 (34, 36)	35 (34, 36)	34 (33, 35)	< 0.05*
***neonatal outcome***s	918	282	36	
Stillbirth, n (%)	1 (0.1)	4 (1.4)	0 (0.0)	< 0.05*
Fetal distress, n (%)	17 (1.9)	4 (1.4)	4 (11.1)	< 0.05*
Neonatal asphyxia, n (%)	10 (1.1)	7 (2.5)	2 (5.6)	< 0.05*
Neonatal death, n (%)	0 (0.0)	0 (0.0)	0 (0.0)	Na
NICU, n (%)	207 (22.6)	73 (26.3)	12 (33.3)	0.17
MSAF, level, n (%)				< 0.05*
No	824 (90.1)	215 (77.9)	23 (63.9)	
Type I (yellow)	57 (6.2)	30 (10.9)	1 (2.8)	
Type II (green)	18 (2.0)	11 (4.0)	7 (19.4)	
Type III (thick)	16 (1.7)	20 (7.2)	5 (13.9)	
Birth height (cm), median (IQR)	46 (44, 47)	45 (43, 47)	43 (42, 45)	< 0.05*
Birth weight (g), median (IQR)	2300 (2040, 2550)	2240 (1965, 2460)	1980 (1747, 2122)	< 0.05*
Birth weight (g), level, n (%)				< 0.05*
< 2500	633 (69.3)	218 (78.1)	34 (94.4)	
[2500-4000]	280 (30.7)	61 (21.9)	2 (5.6)	
> 4000	0 (0.0)	0 (0.0)	0 (0.0)	
Gestational age [week], level, n (%)				< 0.05*
[0,28)	0 (0.0)	0 (0.0)	0 (0.0)	
[28,32)	38 (4.1)	16 (5.7)	0 (0.0)	
[32,34)	78 (8.5)	37 (13.2)	12 (33.3)	
[34,35)	116 (12.6)	34 (12.1)	10 (27.8)	
[35,36)	206 (22.4)	96 (34.2)	14 (38.9)	
[36,37)	290 (31.6)	74 (26.3)	0 (0.0)	
[37, Inf)	190 (20.7)	24 (8.5)	0 (0.0)	

*ICP* Intrahepatic cholestasis of pregnancy, *TBA* Total bile acid, *CS* Cesarean section, *VD* Vaginal delivery, *PTB* Preterm birth, *NICU* Neonatal Intensive Care Unit, *MSAF* Meconium-stained amniotic fluid

**Fig. 1:**
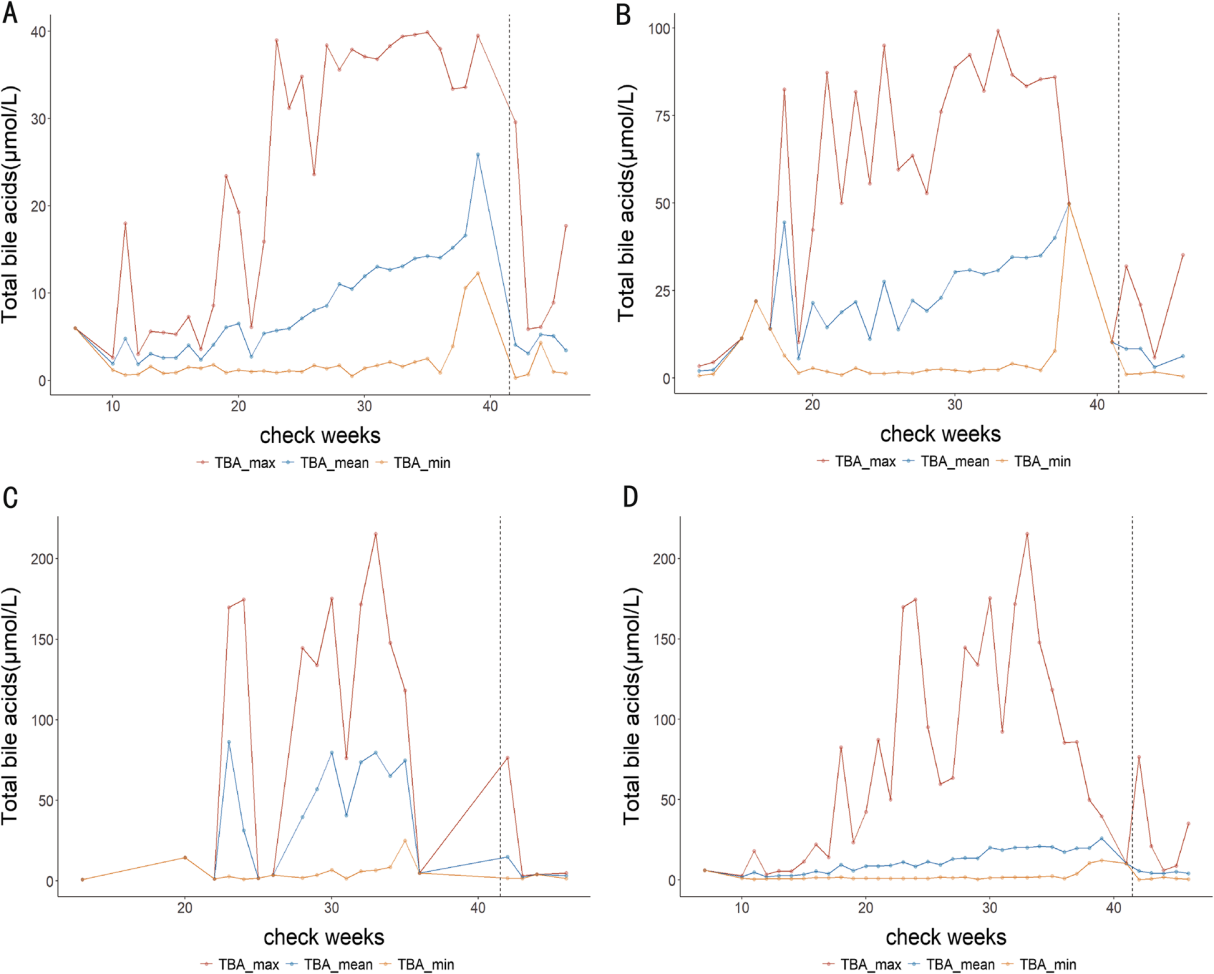
Dynamic TBA variation in twin pregnancies with ICP at various TBA levels. **A** Twin pregnancies with 10 ≤ TBA < 40 μmol/L. **B** Twin pregnancies with 40 ≤ TBA < 100 μmol/L. **C** Twin pregnancies with TBA ≥100 μmol/L. **D** All included twin pregnancies. Three different colored lines are used to depict the dynamic fluctuation of TBA in twin pregnancies with ICP during pregnancy and 5 weeks after delivery. The red line represents the maximum levels of TBA of included patients, the blue line represents the mean levels of TBA of included patients, and the yellow line represents the minimum levels of TBA of included patients

The dynamic fluctuation of TBA-max, TBA-mean, and TBA-min during the entire pregnancy and 5 weeks postpartum at different categories of TBA levels is depicted in Fig. 1. Figure 1-A shows the dynamic fluctuation of twin pregnancies with 10 ≤ TBA < 40 μmol/L, Fig. 1-B shows twin pregnancies with 40 ≤ TBA < 100 μmol/L, Fig. 1-C shows twin pregnancies with TBA ≥100 μmol/L, and Fig. 1-D shows the dynamic fluctuation of all included twin pregnancies. Our data indicate that the peak TBA levels could dynamically change during the entire pregnancy and could quickly increase near term for various TBA levels, even with treatment. However, high TBA levels frequently recovered to normal between 1 and 5 weeks after birth.

**Fig. 2:**
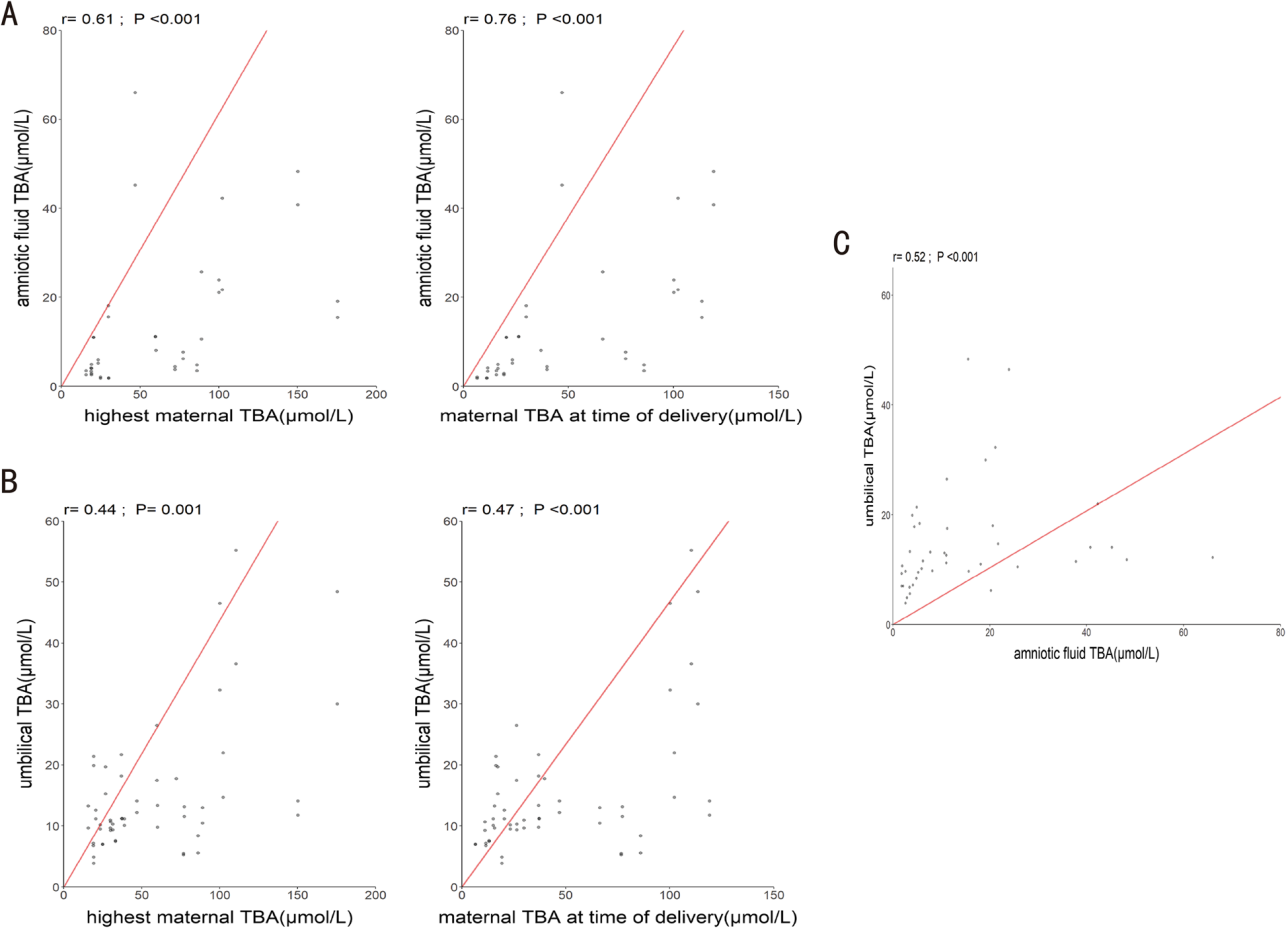
TBA levels in maternal serum, umbilical venous blood, and amniotic fluid with Pearson correlation scatterplots. **A** The ordinate of Fig. 2-A represents TBA levels in the amniotic fluid, Fig. 2-A1 the maximum serum TBA levels (*r =* 0.61, *P <* 0.05), and Fig. 2-A2 the TBA levels upon delivery (*r =* 0.76, *P <* 0.05). **B** The ordinate of Fig. 2-B shows TBA levels in the umbilical venous blood, Fig. 2-B1 shows the highest serum TBA levels (*r =* 0.44, *P <* 0.05), and Fig. 2-B2 shows TBA levels at delivery (*r =* 0.47, *P <* 0.05). **C** The correlation of TBA levels in umbilical venous blood and amniotic fluid (*r =* 0.52, *P <* 0.05) is shown in Fig. 2-C. Significant correlations have a *P* value of less than 0.05

A correlation analysis was performed using matched TBA levels from maternal serum, umbilical venous blood, and amniotic fluid samples from 33 twin pregnancies. Maternal TBA levels at their peak over the entire pregnancy (*r =* 0.61, *P <* 0.05) and upon birth (*r =* 0.76, *P <* 0.05) were positively correlated with the TBA levels in the amniotic fluid (Fig. 2-A). Similarly, maximum maternal TBA levels (*r =* 0.44, *P <* 0.05) and TBA levels at delivery (*r =* 0.47, *P <* 0.05) were positively correlated with TBA levels in the umbilical cord blood (Fig. 2-B). TBA levels in umbilical cord blood and amniotic fluid were similarly found to have a significant and positive correlation (*r =* 0.52, *P <* 0.05) (Fig. 2-C).

**Fig. 3:**
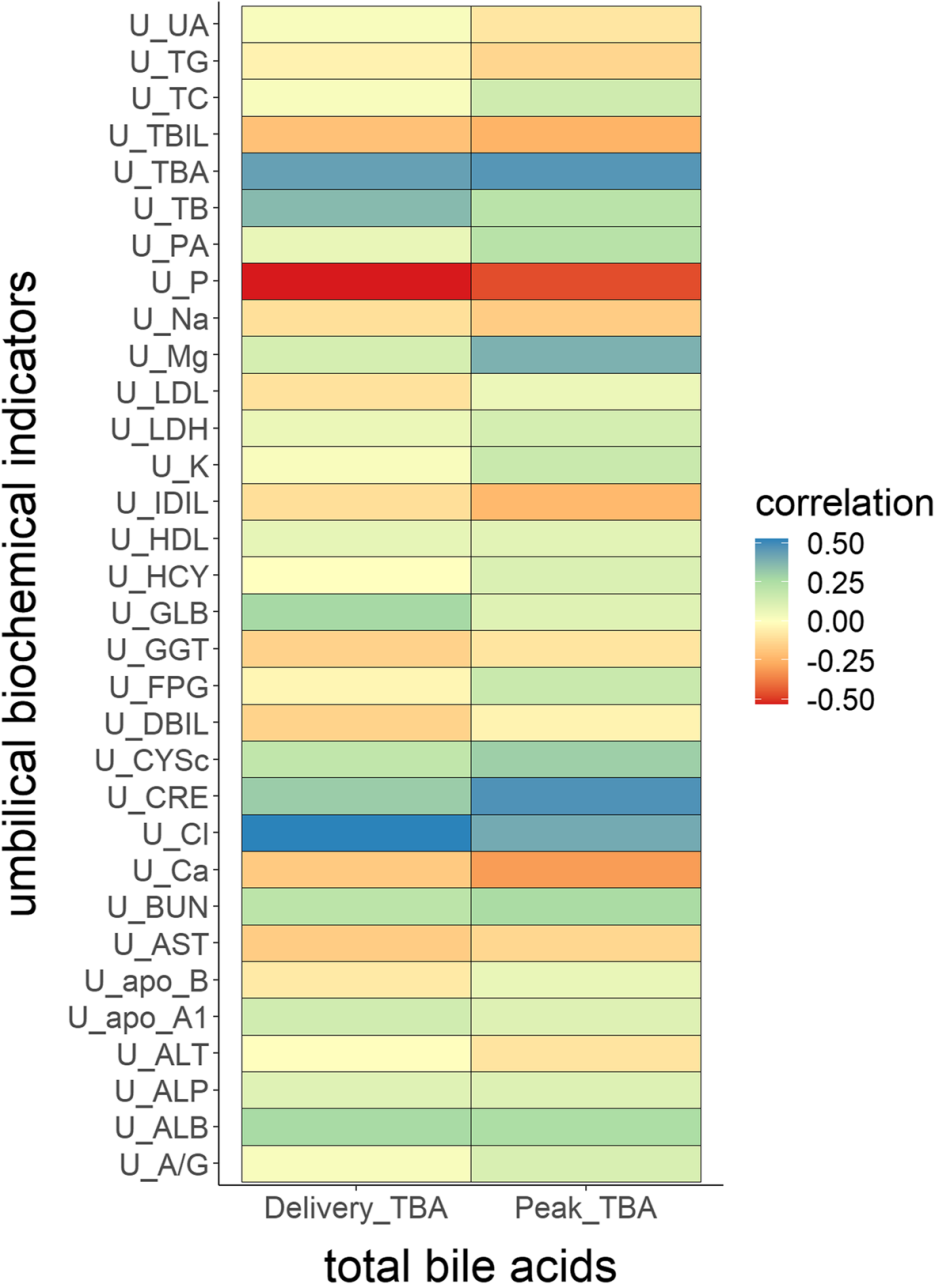
Pearson correlation matrix of maternal serum TBA levels and biochemical markers in umbilical venous blood. The R package “corrgram” was used to create the Pearson correlation matrix: Create a Correlogram (Version 1.13). Using the color map, the Pearson correlation value is color-coded. Positive correlations appear in blue, while negative correlations appear in red. The circle size and color intensity are proportionate to the correlation coefficients. On the right side of the correlogram, the legend color shows the correlation coefficients and the corresponding colors. Correlations with a *P* value of < 0.05 are considered significant in the above graph. Figure 3‘s ordinate depicts biochemical indications of umbilical venous blood, whereas the abscissa represents the highest TBA levels and TBA levels upon delivery

The Pearson parametric correlation test was performed to generate a correlation matrix using all maternal serum maximum TBA levels, TBA levels at delivery, and umbilical cord blood biochemical indicators of these 33 twin pregnancies (Fig. 3). Our findings revealed that umbilical cord blood TBA (*r =* 0.47, *P <* 0.05), CRE (*r =* 0.48, *P <* 0.05), Cl (*r =* 0.41, *P <* 0.05), and Mg (*r =* 0.39, *P <* 0.05) had a significant positive correlation with the maximum maternal serum TBA levels, whereas umbilical cord blood P (*r =* − 0.47, *P <* 0.05) had a significant negative correlation with the maximum TBA levels. The TBA (*r =* 0.44, *P <* 0.05), Cl (*r =* 0.53, *P <* 0.05), TB (*r =* 0.36, *P <* 0.05), GLB (*r =* 0.27, *P <* 0.05), and ALB (*r =* 0.27, *P <* 0.05) levels in umbilical cord blood had a significant positive correlation with maternal serum TBA levels at delivery. A significant negative association was observed between P (*r =* − 0.53, *P <* 0.05) in umbilical cord blood and serum TBA levels at delivery.

## Discussion

Twin pregnancies with ICP had a higher risk of CS, PTB, fetal distress, and NICU admission than singleton pregnancies with ICP. Higher TBA levels were linked to a higher risk of these adverse perinatal outcomes. Furthermore, we discovered a significant positive correlation between maternal serum, amniotic fluid, and umbilical cord blood TBA levels. Similarly, maternal TBA levels were significantly correlated with umbilical cord blood TBA, TB, GLB, ALB, CRE, Cl, and Mg.

Elderly maternal age, a family history of ICP, ICP in a previous pregnancy, and multiple pregnancies have been linked to an elevated risk of ICP [10, 24, 25]. ART, such as IVF-ET and/or ICSI, is associated with an increased risk of PPROM and ICP in twin pregnancies [5, 26]. Similarly, in our study, we discovered that the incidence of a history of ICP and IVF-ET was higher in twin pregnancies than in singleton pregnancies with ICP.

ICP is associated with increased rates of adverse perinatal outcomes [27, 28]. The rates of perinatal mortality, MSAF, fetal distress, and spontaneous PTB were found to be 3.5-11%, 27-45%, 14-22%, and 30-44% in ICP, respectively [29]. Effective fetal surveillance that can lower the risk of adverse fetal outcomes is currently lacking, and few studies have examined twin pregnancy and ICP. In our research, we discovered that twin pregnancies with ICP had a greater rate of CS, PTB, fetal distress, and NICU admission than singleton pregnancies with ICP, implying that ICP is more severe in twin pregnancies than in singletons.

The most lethal adverse perinatal complication of ICP is sudden stillbirth [30]. Stillbirth risk was higher in all cases of ICP [9], especially in those with peak serum TBA ≥ 100 μmol/L [28]. Elevated TBA could cause vasoconstriction disorders in the placental chorionic vessels and umbilical vein [5, 8, 10], which could be a factor in the increased risk of fetal distress, asphyxia, and sudden stillbirth in ICP [3, 5, 31]. ICP with TBA ≥ 100 μmol/L causes 6.8% of prenatal deaths [32]. Most stillbirths of singletons with ICP occur after 37 weeks of pregnancy [33], whereas stillbirths occur at an earlier gestational age in twin pregnancies with ICP. Stillbirth occurs in approximately 3.9% of twin pregnancies with ICP, according to Liu et al. [34]. In our study, the rate of stillbirth in twin pregnancies with ICP was 0.4% (5/633). Our hospital’s aggressive perinatal care management strategies, which were implemented to prevent stillbirth, are responsible for the reduced incidence of stillbirth in twin pregnancies with ICP.

The main concern of ICP management is stillbirth. The perinatal management of twin pregnancies with ICP is challenging for the obstetrician. Given the higher risk of fetal death, different guidelines recommend active management, such as enhanced monitoring and early delivery, to reduce the risk of fetal complications [5], which in turn increases the incidence of iatrogenic PTB [28]. The increased rate of iatrogenic PTB, especially in twin pregnancies, may be due to the active management of ICP. Similarly, our findings corroborated similar conclusions. We discovered that the rate of stillbirth was 0.4% in twin pregnancies with ICP, while the rate of PTB was 82.6%.

In twin pregnancies, ICP is a risk factor for PTB. However, the optimal time of delivery for women with twin pregnancies and ICP has not yet been determined. The reduction in stillbirths needs to be balanced with with the morbidities of prematurity and other perinatal problems. Based on our center’s experience, close antenatal monitoring and active and individual perinatal management are needed. Moreover, the timing of delivery should be discussed on an individual basis according to the known perinatal risk and benefits of available management options.

Our findings also suggest that peak TBA levels may fluctuate throughout pregnancy and may rise rapidly near term, even with medication therapy. Maximum TBA levels were linked to an increased risk of stillbirth in both drug-treated and untreated ICP [28]. Hence, the close and regular monitoring of serum TBA levels is essential; additionally, once ICP is diagnosed, repeating the TBA test until delivery is recommended to help guide the perinatal management of ICP, particularly in severe cases such as twin pregnancies with ICP.

In ICP, UDCA has been shown to relieve maternal pruritus, lower serum TBA levels, and improve liver function [10, 24, 35]. UCDA treatment was provided to all study participants in ours study. Furthermore, we discovered that high TBA levels reverted to normal levels almost 1 to 5 weeks after birth and that liver function returned to normal 4 to 6 weeks thereafter.

Brouwers, L. et al. [22] discovered a positive correlation between maternal serum TBA levels at diagnosis and delivery and umbilical cord blood TBA levels, indicating that TBA can be transported across the placenta. Similarly, we explored the correlations between maternal serum, umbilical cord blood, and amniotic fluid TBA levels in twin pregnancies with ICP. Our findings revealed a strong positive correlation between maternal serum, amniotic fluid, and umbilical cord blood TBA levels. TBA levels in maternal serum, umbilical cord blood, and amniotic fluid have all been found to be higher, which could be due to maternal-fetal circulation. As a result, we hypothesize that TBA is transported through the placenta and is involved in the utero-placental-fetal circulation.

Changes in bile salt and electrolyte transport processes, as well as lipid structure, may be involved in the etiology of ICP, according to Reyes H, et al. [36]. However, the linked etiological mechanism has not been investigated since then. TBA, TB, ALB, GLB, CRE, Cl, and Mg levels in umbilical cord blood had a strong positive correlation with maternal serum TBA levels in our study. This finding may provide some scientific evidence and direction for the etiological mechanism study of bile salt and electrolyte transport pathways in ICP.

An advantage of the current study is that it was one of the largest cohort studies in China focusing on the effect of different categories of TBA levels on the perinatal outcomes of twin pregnancies with ICP. This cohort was studied using the modern active management strategy, as well as dynamic and continuous testing of liver function and TBA levels during pregnancy. Second, we were the first to investigate the link between maternal serum, umbilical cord blood, and amniotic fluid. Finally, we are the first to investigate the link between maternal serum TBA levels and umbilical cord blood electrolyte and bilirubin levels. Our findings point to new directions for research and a better understanding of the etiological mechanisms of bile salt and electrolyte transport pathways in ICP. However, because this work was a single-center retrospective study, it has limitations. A large-scale, multicenter cohort study is necessary in the future to investigate alternative diagnostic and therapeutic interventions in ICP.

## Conclusions

In conclusion, twin pregnancies with ICP in our study had a higher risk of CS, PTB, fetal distress, and NICU admission than singleton pregnancies with ICP, which was related to higher TBA levels. TBA is involved in uterus-placenta-fetal circulation and can be transferred through the placenta.

## Data Availability

The datasets used and/or analyzed during the current study are available from the corresponding author on reasonable request.

## Abbreviations

ICP: Intrahepatic cholestasis of pregnancy
TBA: Total bile acid
CS: Cesarean section
PTB: Preterm birth
NICU: Neonatal intensive care unit
MSAF: Meconium-stained amniotic fluid
RDS: Respiratory distress syndrome
ALT: Alanine aminotransferase
AST: Aspartate aminotransferase
IVF-ET: In vitro fertilization and embryo transfer
VD: Vaginal delivery
DBIL: Direct bilirubin
IDIL: Indirect bilirubin
GGT: γ-glutamyltransferase
ALB: Albumin
GLB: Globulin
A/G: Albumin/globulin
TC: Total cholesterol
TG: Triglyceride
HDL: High-density lipoprotein
LDL: Low-density lipoprotein
ALP: Alkaline phosphatase
LDH: Lactate dehydrogenase
PA: Prealbumin
FPG: Fasting plasma glucose
apo A1: Apolipoproteins A1
apo B: Apolipoproteins B
HCY: Homocysteine
Na: Sodium
P: Phosphorus
Mg: Magnesium
K: Kalium
Cl: Chlorine
Ca: Calcium
NST: Nonstress test
IVH: Intraventricular hemorrhage
MCMA: Monochorionic monoamniotic
MCDA: Monochorionic diamniotic
DCDA: Dichorionic diamniotic
